# Failed Emergence After Pediatric Epilepsy Surgery: Is Propofol-Related Infusion Syndrome to Blame?

**DOI:** 10.7759/cureus.19414

**Published:** 2021-11-09

**Authors:** Tara M Doherty, Catherine Gruffi, Philip Overby

**Affiliations:** 1 Pediatric Anesthesiology, Westchester Medical Center, Valhalla, USA; 2 Pediatric Neurology, Westchester Medical Center, Valhalla, USA

**Keywords:** rhabdomyolysis, fatty acid oxidation, mitochondrial disease, neuromonitoring, total intravenous anesthesia, neurosurgery for epilepsy, lactic acidosis, propofol infusion syndrome

## Abstract

Propofol infusion syndrome was first reported in the literature by Bray in 1998. He described a series of fatal outcomes after a presenting constellation of symptoms observed in pediatric patients who had received prolonged propofol infusions. Profound metabolic acidosis and bradycardia are the disease’s hallmark features, which can further develop expeditiously to rhabdomyolysis, renal failure, and heart failure. It has been subsequently theorized that a triggering mechanism or a precipitating factor sets up the progressive physiologic spiral which can ensue. The name of the disease was expanded to Propofol Related Infusion Syndrome (PRIS), as propofol alone was no longer considered the culprit. The disease process is rare and can present with an insidious onset in some cases, causing much speculation of whether there is a proper grasp of the disease entity in its entirety as currently reported. The case discussed in this article depicts an adverse neurologic outcome following a craniotomy for temporal lobectomy in a child with lesional epilepsy. Since there was no obvious causative factor for these findings, PRIS became a diagnosis that was robustly discussed among the involved services.

## Introduction

Propofol-related infusion syndrome (PRIS) is a rare and potentially fatal syndrome characterized by severe metabolic acidosis, rhabdomyolysis, renal failure, and heart failure [1]. Bray and colleagues initially reported PRIS as a result of increased morbidity and mortality in pediatric intensive care patients receiving long term (>48 hours) and high dose (>4mg/kg/hr or 67 mcg/kg/min) propofol infusions [2]. It is theorized that an underlying condition may be a precipitating factor of PRIS, mainly when an inflammatory illness or an acute neurologic injury is present [1]. Other risk factors include carbohydrate depletion as well as the exogenous use of glucocorticoid and catecholamines. Propofol was approved for use in 1989, and since its introduction, it has been one of the most commonly used anesthetic agents for both the induction and maintenance of anesthesia. Propofol possesses desirable sedative, hypnotic, and anxiolytic properties with a short half-life, making it ideal for intubated patients. Additionally, propofol serves as a neuroprotective agent because of its ability to reduce intracranial pressure. These attributes explain the medication’s early and widespread adoption in intensive care settings. The Adverse Event Reporting System of the FDA reported multiple deaths from non-procedural use of propofol in both children and adults between 1989 and 2005, prompting an immediate change in long-term sedation practice. While the exact mechanism behind propofol infusion syndrome is not fully elucidated, it is a widely accepted theory that the syndrome bears a striking resemblance to the symptomatology of patients with mitochondrial disease experiencing significant metabolic stressors [3]. Patients with mitochondrial disorders have a defect in the mitochondria function and, therefore, impaired adenosine triphosphate production (ATP) generation.

The literature documented that patients with an underlying diagnosis of a mitochondrial myopathy disorder should not receive propofol infusions. In contrast, induction doses of propofol are not considered to increase risk [4]. The organs most vulnerable to a deficiency of ATP production include those with high metabolic demands - the brain, heart, and skeletal muscle [5]. If glucose is not available, the body will alternatively use free fatty acids, and this process, in turn, triggers the release of cortisol and epinephrine, therefore enhancing lipolysis. This alternative energy source will produce substrates used in the citric acid or Krebs cycle, ketone bodies. Propofol interferes with this alternative energy process that is known as the beta-oxidation of free fatty acids. This interference causes cellular hypoxia, a reduction in ATP production, and the accumulation of fatty acids in the liver and other end organs [6]. A metabolic acidosis ensues as these cellular metabolic processes are interrupted, and increased amounts of lactic acid are produced from anaerobic metabolism. Wolf et al. published a landmark paper in 2001 in The Lancet, theorizing that propofol specifically increases malonylcarnitine, which inhibits the activity of carnitine palmitoyltransferase 1 (CPT1), an essential enzyme responsible for converting long-chain fatty acids to acylcarnitine, thereby preventing long-chain fatty acids from entering into the mitochondrial membrane. Activated but not oxidized fatty acids will accumulate in the mitochondrial membrane and prevent proper functioning of the respiratory chain (and therefore decrease ATP production) [7]. Propofol’s ability to prevent the mitochondrial energy production process could be devastating, especially in increased energy demands such as sepsis or significant inflammation. 

## Case presentation

A 55kg, 11-year-old male underwent a right craniotomy and frontal lobectomy for resection of a seizure focus identified by prior intraoperative subdural EEG electrode grid application four days prior. The patient’s previous history was significant for pansinusitis complicated by right holohemispheric subdural empyema and thrombosis of the anterior third of the superior sagittal sinus in January 2019. This was treated with antibiotics and a right frontal craniotomy. In April of 2020, he developed seizures that were fulminant in presentation and rapidly became intractable. Seizure semiology included poor attention at school, body stiffening episodes, and periods of “spacing out.” The increase in seizure frequency had resulted in worsening academic performance (A to C student), blunting of effect, inability to continue with soccer, and impaired peer relationships. He was scheduled for right frontal lobe resection for focal, lesional epilepsy. 

On the morning of surgery, he received his scheduled valproic acid and levetiracetam but experienced breakthrough seizure activity immediately prior to surgery. Therefore, he was transferred from the Pediatric Intensive Care Unit to the operative suite urgently. The patient was induced with rocuronium and propofol for endotracheal intubation. Maintenance included total intravenous anesthesia (TIVA) with propofol and remifentanil infusions in order to accommodate the need for intraoperative neuromonitoring. The case proceeded uneventfully for 12 hours, and approximately one hour prior to the conclusion of surgery, the propofol and remifentanil infusions were discontinued. The total propofol dose throughout the case was approximately 2500 mg. Sevoflurane was initiated at 0.5 MAC in order to expedite emergence. Of note, these actions correlated with the conclusion of neuromonitoring and the beginning of dural closure. During the closure, the patient exhibited gross signs of full-body rigors. In the absence of neuromonitoring, seizure activity could not be confirmed or refuted by that diagnostic modality. At the request of the neurosurgeon, 2 mg of midazolam and 500 mg levetiracetam were given. Despite the cessation of all anesthetics for almost one hour, the patient failed to exhibit spontaneous respiratory effort or response to oral and tracheal suctioning. It also appeared that the patient had a downward gaze of his eyes, but pupillary reflexes were intact. He was brought directly from the operating room to CT to identify a possible post-surgical cause for his delayed emergence. 

CT revealed left to right midline shift into the surgical bed with diffuse loss of grey-white differentiation thought to reflect cerebral and cerebellar edema. The surgeon performed a bi-frontal craniotomy for reexploration based on these findings, which did not reveal a definitive cause. After the surgery, the skull fragment was not replaced in order to accommodate for swelling. The patient’s neurologist was consulted in the OR, and a loading dose of 1000 mg of intravenous fosphenytoin was recommended and administered. The patient remained hemodynamically stable throughout both anesthetics. The patient was transferred to the PICU with plans to maintain deep sedation, ICP monitoring, and continued aggressive seizure prophylaxis for at least 48 hours or until brain edema decreased. Results of an MRI without contrast obtained later that evening included “extensive cerebral and cerebellar edema without evidence for cytotoxic edema. The possibility of toxic or metabolic etiology is favored, florid posterior reversible encephalopathy syndrome (PRES) could also be considered”. The patient had an uneventful ICU course; no observed seizure activity, continuous negative EEG, normal neurologic exams, and was extubated on a postoperative day four after sedation with fentanyl and midazolam infusions weaned, and extubation criteria met. Upon discharge, a non-focal neurologic exam was elicited. The patient exhibited no neurologic sequelae at subsequent outpatient follow-up visits with his neurologist with a significant improvement from his baseline symptoms and was free to resume all activities.

## Discussion

Pro propofol-related infusion syndrome

This is a case of an 11-year-old boy with medically refractory, focal, lesional epilepsy who developed marked encephalopathy intraoperatively. Specifically, he had failed emergence from anesthesia, and imaging was notable for marked cerebral edema in the cortex and basal ganglia with a symmetrical appearance. It needs to be stated that while this patient lacked classic manifestations of PRIS, he did possess features that could be representative of a more subtle or atypical presentation. Given the combination of the patient’s repeated exposure to high doses of propofol, transient elevations in serum lactate, postoperative clinical neurologic status, and abnormal MRI imaging, a metabolic etiology was given high consideration. In particular, the pediatric neurology service proposed propofol-related infusion syndrome to explain the clinical and radiological findings for the following reasons.

Prolonged propofol dosing

The patient underwent a lengthy surgery with a propofol-based anesthetic twice within four days. During the initial procedure of subdural grids, the propofol infusion was dosed at 200 mcg/kg/min for 300 minutes duration and other components of the TIVA regimen. He then received a propofol infusion for the scheduled removal of the grids and frontal lobectomy four days later. This procedure was much longer, and the patient received an average propofol dose of 107 mcg/kg/min for 420 minutes. The propofol dosing was well above the documented threshold for PRIS [2]. It is well described in the literature that high dose propofol infusions are known to contribute to PRIS. According to the MedWatch database, 68% of the cases of PRIS had documented infusions exceeding 83 mcg/kg/min or 5mg/kg/hr, and 54% of the cases had received infusions of over 48 hours [8].

Toxic brain edema

**Figure 1:**
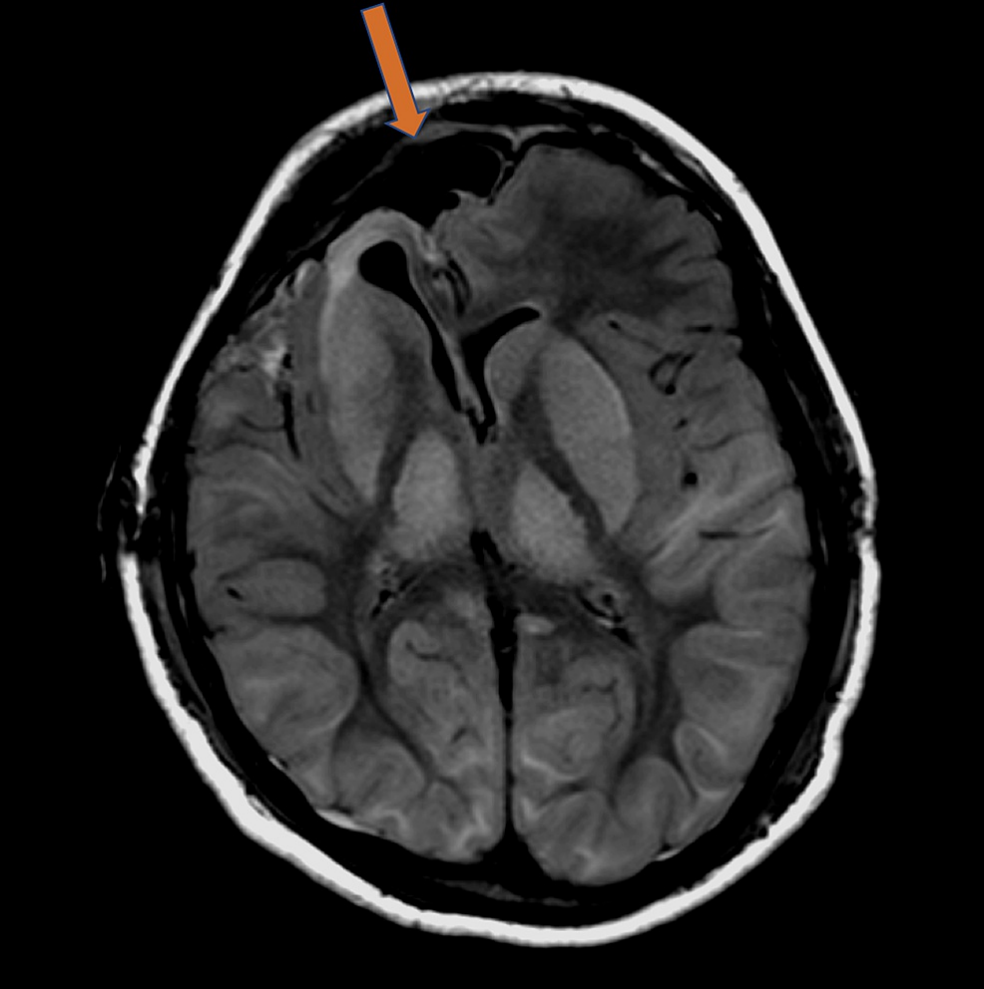
FLAIR image, postoperative day 0

This patient’s clinical findings are limited almost exclusively to significant nervous system deficiencies with failed emergence, as well as markedly abnormal brain imaging. This patient’s findings on MRI are most consistent with a metabolic process, including those listed in a recent review of PRIS [9]. MRI with Fluid-attenuated inversion recovery (FLAIR) sequence revealed significant, symmetric inflammation of the cerebral cortex, particularly parietal, occipital, and posterior temporal lobes. A FLAIR sequence is an imaging modality that removes the cerebrospinal fluid signal, resulting in improved visualization of the grey and white matter of the brain tissue, allowing for better recognition of subtle changes in the cortex and subcortical regions [10]. Brain MRI was obtained after surgery showing an extensive parenchymal signaling abnormality (see Figure 1).

Additionally, there was T2 prolongation involving the basal ganglia and thalami, large regions of the cerebral cortex (most evident in the parietal, occipital, and posterior temporal lobes), and the cerebellum. The T2 prolongation extended to the peripheral subcortical white matter. Based on these MRI findings, posterior, reversible, encephalopathy syndrome or PRES was given a high position on the differential. PRES is a clinico-radiographical syndrome characterized clinically by headaches, seizures, and altered mental status and radiographically by acute symmetric white matter edema typically of the posterior and parietal lobes on MRI imaging [10]. Potential causality of PRES includes hypertension (resulting in cerebral hyperperfusion), sepsis, autoimmune disorder, and cytotoxic medications [11]. Two long propofol anesthetics within such short time proximity in the face of an acute neurologic injury, as demonstrated on MRI, is a possible indication that the patient experienced PRES as a result of PRIS.

Concurrent use of valproic acid and propofol

In a retrospective analysis, it was discovered that the patient possessed two potential risk factors for PRIS: low serum albumin and the recent use of valproic acid. The patient’s albumin values ranged from 2.1-2.7 g/dl prior to the lobectomy surgery. These values are well below the reference range for albumin (3.4-4.8 g/dl). Valproic acid competitively inhibits the cytochrome p450 isoforms clinically relevant, binds to albumin avidly, and frequently displaces other agents [12]. We speculate that the low albumin combined with concomitant valproic acid use may have resulted in higher than expected free serum propofol levels and associated PRIS. In other words, the effective amount of free propofol may have been elevated due to decreased protein binding of propofol: both from low overall serum albumin and displacement from the protein by valproic acid. Alternative explanations remain possible. While carnitine and acylcarnitine profiles were evaluated and were normal, the further occult metabolic disease cannot be excluded. 

Con propofol-related infusion syndrome

This case describes a relatively healthy young boy with a prolonged emergence following an uneventful frontal craniotomy for seizure focus resection. Here we explore the differential causes for this outcome with the primary question: is this propofol infusion syndrome? The anesthesiology team did not feel that the symptom presentation could be attributed to PRIS, which is addressed by listing the syndrome’s features and how each was not clinically relevant to this case.

Metabolic acidosis

Metabolic acidosis is a characteristic and early sign of propofol infusion syndrome secondary to excessive lactic acid accumulation from either increased production or impaired elimination. Lactic acid, a byproduct of anaerobic metabolism, increases from a stultification in the intracellular oxidative phosphorylation and the mitochondria’s inability to meet metabolic demands secondary to inhibition of the electron transport chain in muscle tissue. This imbalance can compromise cardiac and skeletal muscle tissue functions [13]. Lactic acidosis is defined as elevated lactate with a pH <7.35 and a bicarbonate < 20mmol/l [14]. Levels of this magnitude would affect pH, contributing to an overwhelming acidosis and, most likely, a high anion gap metabolic acidosis (HAGMA). 

There was no evidence of acidosis in this case. The pH remained within normal levels on all perioperative arterial blood gas evaluations. Lactic acid was mildly elevated at the end of the case at 2.8 mmol/L (pH 7.47) and peaked in the ICU at 3.6 mmol/L (pH 7.44) soon after arrival. Each of these results was accompanied by only a minimal increase in base excess -2.4 mmol/L and -1.9mmol/L, respectively (see Table 1, 2). We feel that these levels are consistent with a restrictive fluid administration regimen that is desirable and routine during large intracranial procedures. Avoidance of excessive fluid administration reduces the incidence of cerebral edema and increased intracranial pressure. Of note, a similar isolated mild elevation in lactate was seen during the patient’s original surgery the year prior. The other potential cause of an elevated lactic acid would be the possibility that the patient was experiencing non-convulsant seizure activity throughout the procedure masked by our anesthetic. However, one would expect again to see higher levels of lactic acid in that case. Lactate can be elevated after a generalized epileptic attack from muscle hypoxia [15]. Neuromonitoring, in this case, included depth-only electrocorticography (ECoG) with only 4 of the 16 leads by the American Clinical Neurophysiology Society protocol following grid placement, which would be too minimal to detect seizures throughout the case reliably. Also, all brain leads were removed prior to closure.

**Table 1:** Intra-operative blood gas analysis during bifrontal craniotomy re-exploration PCO_2_: Partial pressure of carbon dioxide; PO_2_: Partial pressure of oxygen; Fi0_2_: fraction of inspired oxygen;

Blood gas values	Value 1 18:40	Value 2 20:05
pH (7.35-7.45)	7.47	7.44
PCO_2_ (35-45) mmHg	28	32
PO_2_ (80-100) mmHg	223	230
Base Excess (-3-3) mmol/L	-2.4	-1.9
Bicarb (20-26) mEq/L	20.4	21.7
O_2_ saturation	100.2	100.5
Lactate (0.5-2)	2.8	3.6
Hemoglobin (14-18) g/dL	10.1	9.8
Fi0_2_ %	50	50

**Table 2:** Immediate postoperative blood gas analysis in PICU PCO_2_: Partial pressure of carbon dioxide; PO_2_: Partial pressure of oxygen; Fi0_2_: fraction of inspired oxygen

Blood gas values	Value 3 22:00	Value 4 00:30	Value 5 02:30
pH (7.35-7.45)	7.47	7.46	7.41
PCO_2_ (35-45) mmHg	31	33	39
PO_2_ (80-100) mmHg	216	180	110
Base Excess (-3-3) mmol/L	-0.5	-1.9	0.1
Bicarb (20-26) mEq/L	22.6	23.5	24.7
O_2_ saturation	100.4	100.4	100.1
Lactate (0.5-2)	3.3	2.5	1.5
Hemoglobin (14-18) g/dL	10.2	9.4	9.5
Fi0_2_ %	40	40	40

Rhabdomyolysis and renal failure

Rhabdomyolysis is a rare and potentially lethal clinical process whereby the breakdown of skeletal muscle cells results in myoglobinuria and subsequent renal failure. Rhabdomyolysis is a hallmark feature of propofol infusion syndrome. Propofol can cause toxicity and dysfunction of the mitochondria and, therefore, decrease ATP production [1]. Propofol, in susceptible settings, can be responsible for uncoupling oxidative phosphorylation, thereby inhibiting the regular function of the respiratory chain [16]. Additionally, propofol can cause an increase in malonylcarnitine, interfering with the utilization of fatty acids into the mitochondrial membrane, which reduces energy production through catecholaminergic lipolysis [7]. Laboratory confirmation of rhabdomyolysis would be an elevation in creatine kinase (CK) levels. 

There were no signs or symptoms of rhabdomyolysis in this case. Urine output was appropriate in amount and never abnormal or dark in color. Creatine kinase, a biomarker of muscle injury, was not elevated. Potassium measurements were always within normal limits. Neither blood urea nitrogen nor creatinine was elevated, and therefore, there was no suspected renal dysfunction or failure present.

Cardiac arrhythmia and heart failure

Bradyarrhythmia is a hallmark finding in patients with propofol infusion syndrome. The cardiac and skeletal muscles are primarily affected when a defective mitochondrial respiratory chain results in energy utilization problems. The Kam and Cardone paper collected retrospective data on 20 pediatric patients with reported PRIS and found that 6 of these patients developed a right bundle branch block with (‘coved type’) ST-segment elevations in the right precordial distribution consistent with Brugada syndrome [17]. It is postulated that the buildup of the long-chain fatty acids is responsible for these ventricular arrhythmias [16]. Cardiac arrhythmias are considered an early sign of PRIS. The accumulation of long-chain fatty acids can significantly impair the function of the cardiac myocytes and eventually result in congestive heart failure.

There was no cardiovascular instability throughout this case. There were no intraoperative electrocardiographic changes noted. This patient never demonstrated any arrhythmias or bradycardia for the entire perioperative period and was hemodynamically stable throughout.

Cardiac arrhythmia and heart failure

Bradyarrhythmia is a hallmark finding in patients with propofol infusion syndrome. The cardiac and skeletal muscles are primarily affected when a defective mitochondrial respiratory chain results in energy utilization problems. The Kam and Cardone paper collected retrospective data on 20 pediatric patients with reported PRIS and found that 6 of these patients developed a right bundle branch block with (‘coved type’) ST-segment elevations in the right precordial distribution consistent with Brugada syndrome [17]. It is postulated that the buildup of the long-chain fatty acids is responsible for these ventricular arrhythmias [16]. Cardiac arrhythmias are considered an early sign of PRIS. The accumulation of long-chain fatty acids can significantly impair the function of the cardiac myocytes and eventually result in congestive heart failure.

There was no cardiovascular instability throughout this case. There were no intraoperative electrocardiographic changes noted. This patient never demonstrated any arrhythmias or bradycardia for the entire perioperative period and was hemodynamically stable throughout. 

Liver enlargement 

Another common feature of propofol infusion syndrome is hepatic enlargement, often from the accumulation of fatty acids in the liver [18]. There was no documented finding of hepatic enlargement on physical exam or measured by elevated liver enzymes.

Propofol infusion duration

The total duration of the propofol infusion was approximately seven hours. This was not considered atypical for a neurologic procedure of this nature where propofol is customarily run as part of a TIVA in order to achieve adequate neuromonitoring signals. The average propofol dose was 107 mcg/kg/min or 6 mg/kg/hr and was run for 420 minutes in total. The total intravenous anesthetic included remifentanil (range 0.1-0.2 mcg/kg/min) and less than 1mcg/kg of dexmedetomidine boluses throughout the case to create a balanced anesthetic for neurosurgical optimization. No steroids or vasopressor infusions were utilized or needed throughout, potentially predisposing the patient to further insults to mitochondrial functions. There is evidence that supplemental steroid administration can interfere with gene transcription and affect mitochondrial energy production. This is why steroids have been thought to play the role of a priming factor in PRIS [18]. While some of the PRIS case reports discuss relatively short propofol infusion duration, it was found that these patients had congenital mitochondrial defects and therefore were unable to tolerate propofol infusions [4].

Mitochondrial myopathy (pre-existing)

Mitochondrial disorders are genetic conditions that affect the mitochondria of the cells leading to inadequate energy production. The symptom presentation has a wide range of severity and can present at any age [19]. Mitochondrial disorders can be challenging to diagnose and require a high index of suspicion with vague and mild symptomatology. Patients who have a diagnosis of mitochondrial myopathy require additional management precautions in the perioperative period. Because of impaired mitochondrial function, these patients are exquisitely vulnerable to anesthetics, especially propofol. It is important that fasting is minimized and glucose-rich and lactate deficient solutions are initiated early on. It is possible that patients can present for a surgical procedure without a preexisting diagnosis and only be uncovered by a delayed emergence to a routine anesthetic. 

There was no evidence of the patient having a pre-existing mitochondrial disorder in terms of history or symptoms. The patient had also received similar anesthetics in the past. Laboratory testing showed no rise in total carnitine, acylcarnitine, or free carnitine, which would indicate the presence of a disorder of fatty acid oxidation, new or preexisting.

Hypertriglyceridemia

Patients on long-term propofol infusions or those with PRIS would have elevated triglycerides in the blood due to the liver’s inability to properly regulate plasma lipids. Specific features of the syndrome could compound these effects, such as poor tissue oxygenation or low plasma glucose levels, potentially leading to a higher proportion of propofol remaining in the lipid phase and rendering standard doses less effective [20]. The lipemia index was negative, and triglycerides were normal on laboratory analysis for our patient.

## Conclusions

Our patient demonstrated a concerning postoperative presentation with abnormal brain imaging in correlation with a failure to emerge from anesthesia after consecutive propofol-based anesthetics. It is not easy to ascertain if this clinical scenario can be completely attributed to the use of propofol, as discussed in detail throughout this manuscript. While PRIS, under the classic definition, can be refuted in this case, is it possible to eliminate the diagnosis? The exact mechanism of PRIS is still unknown, most likely as a result of its rarity and inability to conduct prospective randomized controlled trials. Clinical features vary, and there are multiple case reports in which the patients may have only demonstrated 1 or 2 symptoms. In a recent literature review, there was no single feature common to all cases, though 80% of children and adults had metabolic acidosis; 75% of children and 63% of adults had EKG changes. It is essential to continue gathering and examining clinical presentations through case reports to establish better a pattern of events attributed to PRIS. PRIS has a potentially fatal outcome, and its association with neurologic injury is not well reported in the literature. It is also essential to understand the exact mechanism of how propofol contributes to the metabolic derangements that occur with PRIS. The main goal should be to continue gathering data, especially in cases where there is a neurologic insult, to make sure we are not overlooking additional criteria that may need to be included for PRIS diagnosis.

## References

[1] Vasile B, Rasulo F, Candiani A, Latronico N (2003). The pathophysiology of propofol infusion syndrome: a simple name for a complex syndrome. Intensive Care Med.

[2] Bray RJ (1998). Propofol infusion syndrome in children. Paediatr Anaesth.

[3] Hemphill S, McMenamin L, Bellamy MC, Hopkins PM (2019). Propofol infusion syndrome: a structured literature review and analysis of published case reports. Br J Anaesth.

[4] Savard M, Dupré N, Turgeon AF, Desbiens R, Langevin S, Brunet D (2013). Propofol-related infusion syndrome heralding a mitochondrial disease: case report. Neurology.

[5] Hsieh V, Krane E, Morgan P (2017). Mitochondrial disease and anesthesia. J Inborn Errors Metab Screen.

[6] Mirrakhimov AE, Voore P, Halytskyy O, Khan M, Ali AM (2015). Propofol infusion syndrome in adults: a clinical update. Crit Care Res Pract.

[7] Wolf A, Weir P, Segar P, Stone J, Shield J (2001). Impaired fatty acid oxidation in propofol infusion syndrome. The Lancet.

[8] Fong JJ, Sylvia L, Ruthazer R, Schumaker G, Kcomt M, Devlin JW (2008). Predictors of mortality in patients with suspected propofol infusion syndrome. Crit Care Med.

[9] Pernicone E, Watal P, Dhar D, Hayes LL, Chandra T (2020). Neuroimaging of propofol infusion syndrome: a case report and review of literature. Cureus.

[10] Casey SO, Sampaio RC, Truwit CL (2000). Posterior reversible encephalopathy syndrome: utility of fluid-attenuated inversion recovery MR imaging in the detection of cortical and subcortical lesions. Am J Neuroradiol.

[11] Fugate JE, Rabinstein AA (2015). Posterior reversible encephalopathy syndrome: clinical and radiological manifestations, pathophysiology, and outstanding questions. Lancet Neurol.

[12] Wen X, Wang JS, Kivistö KT, Neuvonen PJ, Backman JT (2001). In vitro evaluation of valproic acid as an inhibitor of human cytochrome P450 isoforms: preferential inhibition of cytochrome P450 2C9 (CYP2C9). Br J Clin Pharmacol.

[13] Kraut JA, Madias NE (2014). Lactic acidosis. N Engl J Med.

[14] Ne-Hooi WL, Nair P (2013). Propofol infusion syndrome. continuing education in anesthesia. Critical Care & Pain.

[15] Amrein S, Amrein K, Amegah-Sakotnik A, Reist U, Ensner R (2011). Propofol infusion syndrome--a critical incident report highlighting the danger of reexposure. J Neurosurg Anesthesiol.

[16] Anand K, Ramsay MA, Crippin JS (2001). Hepatocellular injury following the administration of propofol. Anesthesiology.

[17] Kam PC, Cardone D (2007). Propofol infusion syndrome. Anaesthesia.

[18] Scheller K, Sekeris CE (2003). The effects of steroid hormones on the transcription of genes encoding enzymes of oxidative phosphorylation. Exp Physiol.

[19] Vento JM, Pappa B (2013). Genetic counseling in mitochondrial disease. Neurotherapeutics.

[20] Fudickar A, Bein B (2009). Propofol infusion syndrome: update of clinical manifestation and pathophysiology. Minerva Anestesiol.

